# Heated egg yolk challenge predicts the natural course of hen’s egg allergy: a retrospective study

**DOI:** 10.1186/s40413-016-0121-4

**Published:** 2016-10-06

**Authors:** Yu Okada, Noriyuki Yanagida, Sakura Sato, Motohiro Ebisawa

**Affiliations:** 1 Department of Paediatrics, Sagamihara National Hospital, 18-1, Sakuradai, Minami-ku, Sagamihara, Kanagawa 252-0392 Japan; 2 Department of Allergy, Clinical Research Centre for Allergology and Rheumatology, Sagamihara National Hospital, 18-1, Sakuradai, Minami-ku, Sagamihara, Kanagawa 252-0392 Japan; 3 Department of Family Medicine, Kameda Family Clinic Tateyama, 4304-9, Masaki, Tateyama, Chiba 294-0051 Japan

**Keywords:** Challenge test, Egg allergy, Food allergy, Specific IgE, Prognosis

## Abstract

**Background:**

Children do not always outgrow hen’s egg allergies in early childhood. Because egg yolks are less allergenic than egg whites, we performed an oral food challenge with heated egg yolk slightly contaminated with egg white (EYSEW OFC) in infants allergic to hen’s egg. We hypothesized that the EYSEW OFC results would predict the egg allergy’s natural course.

**Methods:**

We retrospectively reviewed participants with hen’s egg allergy who underwent their first EYSEW OFC at 12–23 months of age between 2004 and 2010. Participants who passed the first EYSEW OFC were defined as EYSEW-tolerant, and participants who failed the OFC were defined as EYSEW-reactive. Participants who passed the EYSEW OFC underwent an OFC with half of a heated whole egg (WE OFC). Participants who passed a WE OFC were defined to be heated hen’s egg-tolerant. Participants who failed the EYSEW OFC or the WE OFC underwent another OFC at least 6 months later. We compared tolerance to heated hen’s egg at 36 months after the first EYSEW OFC between EYSEW-tolerant and EYSEW-reactive participants. Univariate and multivariate logistic regression analyses were conducted.

**Results:**

Of the 197 included participants (median age: 18.3 months; range: 12.1–23.8 months), 179 (90.9 %) were EYSEW tolerant and 18 (9.1 %) were EYSEW reactive. At 36 months after the first EYSEW OFC, 164 EYSEW-tolerant (91.6 %) and 12 EYSEW-reactive participants (66.7 %) achieved heated hen’s egg tolerance. In the univariate logistic regression analyses, EYSEW-reactive participants (crude odds ratio [OR], 5.5 [95 % confidence intervals [CI], 1.8–16.6]; *p* = 0.003) and those with baseline egg white sIgE levels (crude OR: 3.9 per ten-fold increase [95 % CI, 1.5–10.2]; *p* = 0.005) had greater odds of persistent allergy to hen’s egg at 36 months after the first EYSEW OFC. In a multivariate logistic regression analysis after adjustment for baseline egg white sIgE, EYSEW-reactive participants had greater odds of persistent allergy to hen’s egg than EYSEW-tolerant participants (adjusted OR: 4.6 [95 % CI, 1.5–15.0]; *p* = 0.003).

**Conclusions:**

Classifying infants who are allergic to hen’s egg into EYSEW tolerant and EYSEW reactive groups was useful in determining prognosis.

## Background

The estimated prevalence of egg allergy is 2 % among children aged 1–3 years in the United Kingdom [1] and 1.8 % among children aged 1–5 years in the United States [2]. In Japan, the estimated prevalence of food allergy of any kind is 5–10 % among infants; among infants with food allergy, 44.6–62.1 % of infants are allergic to eggs [3]. Egg allergy prognosis varies in different studies. In some studies, 66–74 % of egg allergy cases were reported to outgrow their allergy by the age of 5 years [4, 5]. In contrast, another study found that only 12 % of egg allergy cases outgrew their allergy by the age of 6 years and 48 % by 12 years [6]. These studies show that the prognosis of egg allergy is not necessarily good.

Oral food challenge (OFC) with baked egg is a practical approach for a better prognosis in these children because baked egg-tolerant children are more likely to resolve their hen’s egg allergies [7, 8]. Leonard et al. reported that participants in an intent-to-treat group underwent a baked egg challenge in which baked egg-tolerant participants were advised to consume a baked egg, for example, in a muffin or waffle. Participants in the comparison group strictly avoided eggs. At the end of the study, 53 % of participants in the intent-to-treat group and 28 % of participants in the comparison group tolerated raw eggs [7]. Peters et al. reported that baked egg-tolerant participants consumed a baked egg at the age of 1 year. By the age of 2 years, egg allergy resolution was higher in baked egg-tolerant participants than in baked egg-allergic participants (56 % and 13 %, respectively) [8]. Many children who are allergic to hen’s eggs tolerate baked eggs [7–9]. However, children who react to baked eggs must avoid hen’s eggs completely.

The effects of egg oral immunotherapy (OIT) using egg powder, scrambled eggs, or raw hen’s eggs as a therapeutic diet have also been reported [10–15]. Burks et al. randomized children with egg allergies to receive egg OIT or placebo treatment. At 10 months, 55 % of participants who received OIT and no participant who received placebo treatment were desensitized. However, during the 10 months, the adverse event rate in the OIT group was higher than that in the placebo group (25.0 % and 3.9 %, respectively) [10]. Furthermore, there are few research institutions that conduct egg OIT.

In cooking, raw egg yolks with slight egg white contamination confer characteristics such as coagulation, emulsification, and foaming, and can be used in hamburger patties and cakes. Because egg yolks are less allergenic than egg whites [16], we initially performed an OFC with heated egg yolks slightly contaminated with egg white (EYSEW OFC) for hen’s egg allergies. Then, an OFC was performed with half of a heated whole egg (WE OFC) among children who demonstrated EYSEW tolerance in the EYSEW OFC. Foods containing one heated egg yolk with slight egg white contamination, from which we removed the majority of the egg white, are low allergenic egg products.

## Methods

### Aim

The purpose of the present study was to retrospectively evaluate if the results of EYSEW OFC in infants who are allergic to hen’s egg could predict the natural course of egg allergies based on our daily practice.

### Participant selection

Eligible participants were children who underwent their first EYSEW OFC at a national allergy centre in Japan at 12–23 months of age between 2004 and 2010, had a history of immediate reactions within 2 h after ingesting eggs, and were positive for egg white-specific immunoglobulin E (sIgE). Participants lost to follow-up in the 36 months after the first EYSEW OFC were excluded.

### Study design

Participants who passed the first EYSEW OFC were defined as EYSEW-tolerant, and participants who failed the OFC were defined as EYSEW-reactive. Participants who passed a WE OFC were defined to be heated hen’s egg-tolerant. We retrospectively compared heated hen’s egg tolerance 36 months after the first EYSEW OFC between EYSEW-tolerant and EYSEW-reactive participants.

**Fig. 1:**
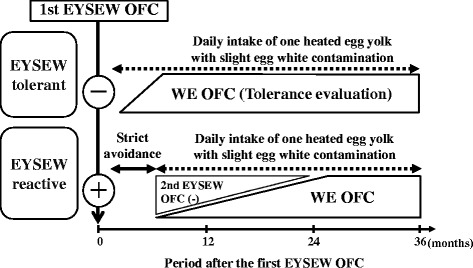
Management of egg allergy based on OFC in EYSEW-tolerant participants and EYSEW-reactive participants. EYSEW: heated egg yolk slightly contaminated with egg white; OFC: oral food challenge; WE: heated whole egg

Participants with egg allergies always underwent EYSEW OFC first. Before participants passed the EYSEW OFC, they strictly avoided egg. Participants who failed the first EYSEW OFC (i.e., EYSEW-reactive participants) underwent another EYSEW OFC at least 6 months later. Participants who passed the EYSEW OFC were advised to consume a pancake or hamburger patty containing one heated egg yolk with slight egg white contamination at home at least twice a week, and underwent a WE OFC. Participants who failed the first WE OFC underwent another WE OFC at least 6 months later (Fig. 1).

### Assessment of baseline characteristics

The attending physician was responsible for diagnoses of hen’s egg allergies, anaphylaxis due to egg, other food allergies, and a history of eczema or asthma. A history of eczema was defined as having had eczema at any time between birth and the first EYSEW OFC.

### Laboratory test

Egg white sIgEs were assessed using the ImmunoCAP assay system (Thermo Fisher Scientific, Uppsala, Sweden) for all participants. The measurement range was 0.35–100 kUA/L, and a measurement > 0.35 kUA/L was considered positive. We did not dilute the serum for measurements >100 kUA/L at that time; therefore, the results were conveniently approximated to 101 kUA/L. The median duration between the laboratory test and first EYSEW OFC was 4.4 months (range: 0.0–13.1 months).

### Oral food challenge protocol

The food used in the hen’s egg OFC was pumpkin cake. For foods used in the OFC with EYSEW, we removed as much raw egg whites as possible from the raw egg yolks. Each challenge food was prepared by mixing the egg yolks with 50 g of pumpkin, which was then heated to 95 °C for 1.5 min in a 1000-watt microwave. For the WE OFC, we used the same procedure, except with one-half of a whole egg.

OFCs were performed openly under physician observation at Sagamihara National Hospital. Until February 2008, the challenge food was administered at 15-min intervals as follows: 1/16, 1/16, 1/8, 1/4, and 1/2 of the total amount, while from March 2008, the challenge food was administered at 30-min intervals as follows: 1/8, 3/8, and 1/2 of the total amount. The OFC was concluded when the child had consumed a quantity of egg sufficient to cause moderate or severe symptoms (generalized urticaria, continuous coughing, moderate or severe abdominal pain, vomiting, or diarrhoea). If mild objective symptoms (localized urticaria or intermittent coughing) appeared during the OFC, the participant was carefully monitored to detect any worsening of symptoms. If the mild objective symptoms disappeared within 30 min, the OFC was continued. When an adverse reaction occurred, necessary medicines (antihistamine, nebulized β2 agonist, steroids, or adrenaline) were administered based on the European Academy of Allergy and Clinical Immunology (EAACI) food allergy and anaphylaxis guidelines [17].

### Allergenicity of the challenge food

The FASTKIT ELISA Ver. II Egg (Nippon Meat Packers, Inc. R&D Centre, Tsukuba, Japan), which detects egg white proteins including ovomucoid and ovalbumin, was used to measure the amounts of egg white protein in one scrambled egg, the pumpkin cake used in the EYSEW OFC, and the pumpkin cake used in the WE OFC. The mean results of the three measurements were 7,400 mg in one scrambled egg, 209 mg in the EYSEW OFC cake, and 2,220 mg in the WE OFC cake. In other words, the amount of egg white protein in the EYSEW OFC cake was 1/35 of one scrambled egg, and that in the WE OFC cake was 3/10 of one scrambled egg.

### Statistical analysis

Differences in baseline characteristics at the first EYSEW OFC were compared between EYSEW-tolerant and EYSEW-reactive participants using Mann-Whitney tests for continuous variables (expressed as the median and range or interquartile range [IQR]) and the chi-squared or Fisher’s exact tests for categorical variables (expressed as the number and percentage).

Univariate and multivariate logistic regression analyses were conducted to determine predictors of persistent allergy to hen’s egg at 36 months after the first EYSEW OFC, resulting in the estimation of odds ratios (ORs) and corresponding 95 % confidence intervals (CIs). The results of the first EYSEW OFC and baseline characteristics were included as potential predictors. Potential predictors that were significant in the univariate analyses at *p* < 0.05 were included in the multivariate logistic regression model. Kaplan-Meier curves were generated to depict changes in the rates of heated hen’s egg tolerance for EYSEW-tolerant and EYSEW-reactive participants. The differences between EYSEW-tolerant and EYSEW-reactive participants were estimated using the log-rank test. SPSS version 20 (IBM Corp., Armonk, NY, USA) was used for all analyses.

## Results

### Baseline participant characteristics

**Fig. 2:**
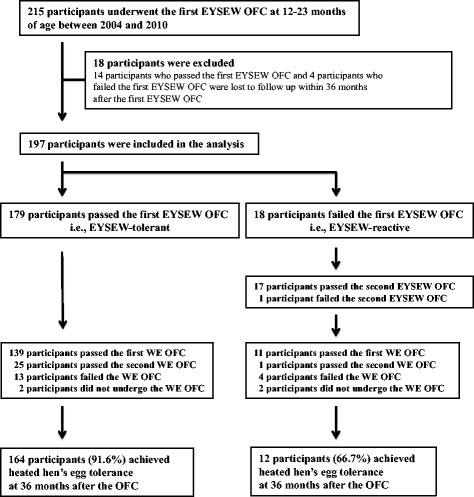
Flowchart of the enrolled participants. EYSEW: heated egg yolk slightly contaminated with egg white; OFC: oral food challenge

Of the 215 participants who underwent their first EYSEW OFC at 12–23 months of age between 2004 and 2010, 18 participants (EYSEW tolerant: *n* = 14; EYSEW reactive: *n* = 4) were lost to follow up within 36 months after the OFC, resulting in 197 participants (median age: 18.3 months; range: 12.1–23.8 months) being included in the analyses (EYSEW tolerant: *n* = 179; EYSEW reactive: *n* = 18). In other words, the positive rate of the first EYSEW OFC was 9.1 % (Fig. 2). When EYSEW-tolerant participants underwent the first EYSEW OFC, 8 EYSEW-tolerant participants had mild objective symptoms. Seven EYSEW-tolerant participants had localized urticaria, and 1 EYSEW-tolerant participant had intermittent coughing. These participants were subsequently able to continue eating a pancake or hamburger patty containing one heated egg yolk with slight egg white contamination at home without any symptoms.

The median duration between the first EYSEW OFC and first WE OFC was 3.7 months among EYSEW-tolerant participants and 18.0 months among EYSEW-reactive participants, except for 2 EYSEW-tolerant participants and 2 EYSEW-reactive participants. Directly after the EYSEW OFC was passed, 4 EYSEW-tolerant participants and no EYSEW-reactive participants underwent the first WE OFC. One EYSEW-reactive participant did not undergo the WE OFC because of failure of the second EYSEW OFC. Based on physician judgment, 2 EYSEW-tolerant participants and another EYSEW-reactive participant did not undergo the WE OFC in the 36 months after the first EYSEW OFC. No baseline characteristic was significantly different between EYSEW-tolerant and EYSEW-reactive participants (Table 1).

**Table 1:** Baseline characteristics of the participants with hen’s egg allergy

Characteristic	All participants (*n* = 197) Med (IQR) or n (%) ^b^	EYSEW tolerant (*n* = 179) Med (IQR) or n (%)	EYSEW reactive (*n* = 18) Med (IQR) or n (%)	*p* value^a^
Age (months), median (range)	18.3 (12.1–23.8)	18.2 (12.1–23.8)	19.0 (12.3–23.4)	0.296
Male	113 (57.4)	101 (56.4)	12 (66.7)	0.402
Anaphylaxis due to hen’s egg	20 (10.2)	19 (10.6)	1 (5.6)	0.431
Other food allergy	122 (61.9)	112 (62.6)	10 (55.6)	0.559
History of eczema	163 (82.7)	146 (81.6)	17 (94.4)	0.144
Asthma	4 (2.0)	4 (2.2)	0 (0.0)	0.680
Total IgE (kUA/L)	67.0 (30.3–181.8)	62.0 (30.0–149.3)	113.0 (39.0–217.3)	0.291
	(*n* = 196)	(*n* = 178)	(*n* = 18)	
Egg white sIgE (kUA/L)	6.6 (2.6–15.3)	5.8 (2.5–14.3)	12.4 (3.2–34.3)	0.139
Egg yolk sIgE (kUA/L)	0.96 (0.15–2.3)	0.96 (0.15–2.2)	0.97 (0.56–3.0)	0.495
	(*n* = 159)	(*n* = 144)	(*n* = 15)	
Ovomucoid sIgE (kUA/L)	2.1 (0.15–7.1)	2.0 (0.15–6.7)	3.0 (0.89–17.7)	0.168
	(*n* = 179)	(*n* = 162)	(*n* = 17)	

A history of eczema was defined as having had eczema at any time between birth and the first EYSEW oral food challenge

*EYSEW* heated egg yolk slightly contaminated with egg white, *IQR* interquartile range, *Med* median, *sIgE* specific IgE

^a^Comparisons between EYSEW-tolerant and EYSEW-reactive participants were conducted using Mann-Whitney tests for continuous variables or the chi-squared or Fisher’s exact tests for categorical variables

^b^Values are reported as the median (interquartile range) or n (%), unless otherwise stated

### Comparison of the natural courses of hen’s egg allergy

At 36 months after the first EYSEW OFC, 164 EYSEW-tolerant participants (91.6 %) and 12 EYSEW-reactive participants (66.7 %) achieved heated hen’s egg tolerance. In the univariate logistic regression analyses, EYSEW-reactive participants had greater odds of persistent allergy to hen’s egg at 36 months after the first EYSEW OFC than EYSEW-tolerant participants (crude OR, 5.5 [95 % CI, 1.8–16.6]; *p* = 0.003). Baseline egg white sIgE levels (crude OR: 3.9 per tenfold increase [95 % CI, 1.5–10.2]; *p* = 0.005), egg yolk sIgE levels (crude OR: 3.6 per tenfold increase [95 % CI, 1.3–9.5]; *p* = 0.012), and ovomucoid sIgE levels (crude OR: 2.9 per tenfold increase [95 % CI, 1.4–5.9]; *p* = 0.005) also resulted in greater odds of persistent allergy to hen’s egg (Table 2).

**Table 2:** Results of logistic regression analyses for persistent allergy to hen’s egg at 36 months

		Univariate		Multivariate 1		Multivariate 2 (*n* = 159^a^)		Multivariate 3 (*n* = 179^a^)	
Variable		Odds ratio (95 % CI)	*p* value	Odds ratio (95 % CI)	*p* value	Odds ratio (95 % CI)	*p* value	Odds ratio (95 % CI)	*p* value
First EYSEW OFC	EYSEW reactive	5.5 (1.8–16.6)	0.003^*^	4.6 (1.4–15.0)	0.010^*^	5.6 (1.5–20.0)	0.009^*^	3.7 (1.1–12.7)	0.037^*^
	EYSEW tolerant	1.0		1.0		1.0		1.0	
Baseline egg white sIgE	per tenfold increase	3.9 (1.5–10.2)	0.005^*^	3.3 (1.3–8.7)	0.013^*^				
Baseline egg yolk sIgE (*n* = 159^a^)	per tenfold increase	3.6 (1.3–9.5)	0.012^*^			3.6 (1.3–10.0)	0.014^*^		
Baseline ovomucoid sIgE (*n* = 179^a^)	per tenfold increase	2.9 (1.4–5.9)	0.005^*^					2.7 (1.3–5.6)	0.009^*^

*CI* confidence interval, *EYSEW OFC* heated egg yolk slightly contaminated with egg white oral food challenge, *EYSEW tolerant* participants who passed the first EYSEW OFC, *EYSEW reactive* participants who failed the first EYSEW OFC, *sIgE* specific immunoglobulin E

^*^ *p* values < 0.05

^a^For multivariate logistic regression analyses, participants who did not undergo baseline egg yolk sIgE (*n* = 38) or baseline ovomucoid sIgE (*n* = 18) were excluded

**Fig. 3:**
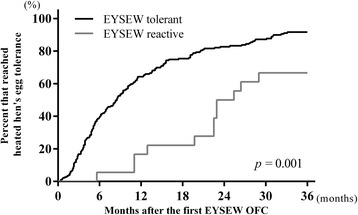
Changes in the rates of heated hen’s egg tolerance. Comparisons between EYSEW-tolerant and EYSEW-reactive participants conducted using log-rank tests are shown. EYSEW OFC: heated egg yolk slightly contaminated with egg white oral food challenge; EYSEW tolerant: participants who passed the first EYSEW OFC; EYSEW reactive: participants who failed the first EYSEW OFC

In the multivariate logistic regression analysis, EYSEW-reactive participants still had greater odds of persistent allergy to hen’s egg at 36 months after the first EYSEW OFC than EYSEW-tolerant participants after adjustment for egg white sIgE (adjusted OR: 4.6 [95 % CI, 1.5–15.0]; *p* = 0.010), after adjustment for egg yolk sIgE (adjusted OR: 5.6 [95 % CI, 1.5–20.0]; *p* = 0.009), and after adjustment for ovomucoid sIgE (adjusted OR: 3.7 [95 % CI, 1.1–12.7]; *p* = 0.037; Table 2). Furthermore, the Kaplan-Meier curves showed that EYSEW-tolerant participants had significantly earlier resolutions of hen’s egg allergies than EYSEW-reactive participants during the 36 months after the first OFC (Fig. 3).

## Discussion

To the best of our knowledge, this is the first study to indicate that EYSEW OFC results in infancy can predict the natural course of hen’s egg allergy in early childhood. Previous studies have reported that predictors of the natural course were egg white sIgE [4–6, 8], skin prick test weal sizes [4, 8], baked egg tolerance [7, 8], other food allergies [6, 8], other atopic diseases [6, 8], and severities of previous reactions [4]. In the present study, after adjustment for egg white sIgE, which is a known predictor in many studies, the EYSEW OFC results remained significant.

The EYSEW OFC can identify children reactive to baked eggs who can begin to consume a portion of a hen’s egg. In the present study, the median egg white sIgE was 6.6 kUA/L and the positive rate in the first EYSEW OFC was only 9.1 %. Comparatively, in baked egg studies, the median egg white sIgEs ranged from 1.7–3.0 kUA/L and the positive rates in baked egg OFCs ranged from 7.3–19.7 % [7–9]. These results suggest that some children who fail a baked egg OFC can pass an EYSEW OFC and start to ingest EYSEW.

Our recipe for the OFC with EYSEW separated the egg yolk from the white before heating to make use of egg yolk characteristics such as coagulation, emulsification, and foaming. EYSEW-reactive participants might have reacted to not only egg yolk protein, but also to the very small amount of egg white protein. As a result of the FASTKIT ELISA Ver. II Egg, the amount of egg white protein in the EYSEW OFC cake was 1/35 of one scrambled egg. If we assess the efficacy of only heated egg yolk, hard-boiled egg yolk is less contaminated with egg white for a challenge food. However, because our recipe can be applied to various dishes, which could lead to a better quality of life for hen’s egg allergic children and their families, a challenge food made from heated egg yolks with slight egg white contamination was used for the OFC with EYSEW in our daily practice.

Ovomucoid is the immunodominant protein fraction in hen’s egg whites [18]. A food matrix of heated egg and wheat flour reduces ovomucoid allergenicity [19]. The EYSEW OFC cake had low allergenicity because of a food matrix of heated egg yolk slightly contaminated with egg white and pumpkin. The WE OFC cake containing one-half of a whole egg had lower allergenicity than one-half scrambled egg because of a food matrix of heated egg and pumpkin.

In Japan, once children who are allergic to hen’s egg are assessed to tolerate heated whole eggs, they can eat lunch boxes with hen’s eggs in kindergarten and school. Therefore, we defined tolerance of a heated whole egg confirmed by a negative result from the WE OFC as the outcome in this study. Further studies are needed to assess whether the results of the EYSEW OFC can predict raw egg tolerance in infants allergic to hen’s egg.

We advised participants who passed the EYSEW OFC consume a pancake or hamburger patty containing one heated egg yolk with slight egg white contamination at home at least twice a week to improve the rate of heated hen’s egg tolerance. Peters et al. reported that baked egg ingestion 1–4 times per month resulted in a higher resolution of raw egg allergy than no consumption [8]. However, it is difficult for us to require participants who passed the EYSEW OFC to avoid heated egg yolk in the real world. In addition to the effects caused by the ingestion of heated egg yolk, EYSEW OFC results may be a predictive factor. A heated egg yolk with slight egg white contamination may be another therapeutic option for baked egg and egg OIT for children with hen’s egg allergy.

Egg protein in baked egg has immunogenicity for tolerance induction in children with egg allergy [7–9]. For baked egg foods, muffins are baked at 176 °C for 30 min in an oven, and waffles are cooked in a waffle maker at approximately 260 °C for 3 min [20]. Because the EYSEW OFC cake is heated to a lower temperature compared to baked egg foods, egg protein in the EYSEW OFC is denatured to a degree that is equal to or less than that of egg protein in baked egg foods. Thus, egg protein in the EYSEW OFC is also expected to have immunogenicity for tolerance induction. For the EYSEW OFC cake, we removed as much egg whites as possible, so it had a low amount of egg white protein. Low dose cow’s milk intake has been shown to increase the food challenge threshold [21–23]. The EYSEW OFC cake probably affects children with egg allergy through the same mechanism.

A limitation of our study is that the amounts of egg white protein in the EYSEW OFC cakes were slightly different depending on the cake, because our recipe for the OFC with EYSEW separated the egg yolk from the white by hand before heating. We performed an OFC with egg powder containing 1/32 of a whole egg recently so that the amounts of egg white protein in the challenge foods are equivalent. Another limitation of our retrospective study includes the lack of a heated WE OFC or raw egg OFC just before the first EYSEW OFC. Some children may have resolved their hen’s egg allergies by the first EYSEW OFC. A third limitation of our retrospective study is that we did not perform the WE OFC at a predetermined age. Two EYSEW-tolerant participants and 2 EYSEW-reactive participants did not undergo a WE OFC. However, almost all of the participants underwent more than one WE OFC for the assessment of tolerance to heated hen’s egg in the 36 months after the first EYSEW OFC.

## Conclusion

Classifying infants who are allergic to hen’s egg into EYSEW-tolerant and EYSEW-reactive groups is useful to determine prognosis. Future randomized controlled trials are needed to determine if the intake of heated egg yolk with slight egg white contamination improves hen’s egg allergies.

## Abbreviations

CI: Confidence interval
EYSEW: Heated egg yolk slightly contaminated with egg white
IQR: Interquartile range
OFC: Oral food challenge
OIT: Oral immunotherapy
OR: Odds ratio
sIgE: Specific immunoglobulin E
WE: Heated whole egg
